# *CHS-2* Is Involved in the Response of *Aedes albopictus* (Diptera: Culicidae) Larvae to Cadmium Stress by Mediating the Formation of the Peritrophic Membrane

**DOI:** 10.3390/insects16111102

**Published:** 2025-10-29

**Authors:** Chen Zhang, Yanjuan Ding, Ruoyun Lan, Min Zhou, Yanrong Chen, Bin Tang, Gexia Qiao, Shigui Wang

**Affiliations:** 1 College of Life and Environmental Sciences, Hangzhou Normal University, Hangzhou 311121, China; 2 State Key Laboratory of Animal Biodiversity Conservation and Integrated Pest Management, Institute of Zoology, Chinese Academy of Sciences, Beijing 100101, China

**Keywords:** chitin synthase, metallothionein, heat shock proteins, antioxidant enzymes, redox reaction

## Abstract

**Simple Summary:**

The exposure of *Aedes albopictus* larvae to cadmium-contaminated water may lead to an increased resistance, thereby complicating vector control efforts. The *CHS-2* gene plays a critical role in the formation of the peritrophic membrane, an essential tissue involved in the larval stress response of *Ae. albopictus*. However, the function of *CHS-2* in mediating the response to cadmium stress remains unclear. In this study, we first exposed *Ae. albopictus* larvae to cadmium and examined *CHS-2* expression levels alongside morphological changes in the peritrophic membrane. Subsequently, RNA interference was employed to silence the *CHS-2* gene, followed by cadmium exposure to assess alterations in larval resistance in the absence of *CHS-2*. Our results elucidate the adaptive responses and underlying mechanisms of *Ae. albopictus* larvae to cadmium exposure, providing a theoretical basis for the utilization of this species in monitoring aquatic environmental contamination.

**Abstract:**

Pollution with heavy metals, such as cadmium (Cd), can significantly affect insect growth, development, and behavior. The midgut is an essential organ for stress response. *Chitin synthase-2* (*CHS-2*) is closely associated with forming the peritrophic membrane (PM). The fourth-instar larvae of *Aedes albopictus* were exposed to varying concentrations of Cd. The results showed that Cd inhibited chitin synthesis and metabolism-related genes, but thickened the midgut PM, indicating that the larvae could respond to Cd stress through the midgut PM. Silencing *CHS-2* by RNA interference resulted more severe vacuolization and malformation of midgut epithelial cells without midgut PM protection. Additionally, there was an intensified redox reaction, upregulated expression of metallothionein (MT) and heat shock proteins 70 (HSP70), and increased activity of antioxidant enzymes at some scattered time points. This study confirms that *CHS-2* is involved in oxidative stress induced by Cd exposure by regulating PM formation. This study also contributes to further understanding the resistance mechanism of *Ae. albopictus* under Cd stress, thereby establishing a theoretical foundation for the future studies of them, which is concerned with the possibility of *Ae. albopictus* as a water environment detection and the control of *Ae. albopictus* based on resistance mechanism.

## 1. Introduction

Nearly all heavy metals occur naturally in an oxidized state in various ecosystems. However, once their concentration surpasses the environmental threshold, these metals threaten ecosystems, resulting in heavy metal pollution. In addition, heavy metals are stable, difficult to degrade, highly toxic and can accumulate in the food chain, making heavy metal pollution harmful and difficult to control. The pollution and degradation of soil and water in China have become increasingly prominent in recent years, exacerbated by rapid industrialization and irrational agricultural practices. Particularly, Cd, Hg, Ni, Cu, and As contamination levels are alarmingly high [1]. Most heavy metals interact with DNA molecules and nuclear proteins to cause DNA damage, leading to apoptosis or cancer [2]. Heavy metals are associated with various diseases and can impair reproductive fitness and neural development [3,4]. Exposure to heavy metals in the environment affects the growth and development of various organisms in the biosphere and indirectly impairs human life and health through accumulation in food chains [5]. Cd, a significant contributor to pollution, has garnered increasing attention in recent years. Cd pollution in China’s rivers is primarily concentrated in the Xiangjiang River Basin and regions such as Jiangsu and Zhejiang, as revealed by a survey [6].

Insects display exhibit a broad spectrum of feeding habits and remarkable adaptability. They are the most abundant and geographically dispersed organisms in the world. When insects reside in environments contaminated with heavy metals, heavy metals can permeate the insect body through respiration, ingestion, and skin penetration, reducing hemolymph energy reserves, disrupting redox balance, and impairing the integrity of cellular structures [7]. Furthermore, heavy metal exposure may enhance the susceptibility of insects to pathogenic microorganisms [8], thereby influencing their growth, development [9,10] and reproductive capabilities [11]. Heavy metal pollution, both direct and indirect, can potentially decrease aquatic insect diversity and trigger the local extinction of insect populations [12]. Insects respond to heavy metal stress through a series of reactions and mechanisms, such as the active deposition and expulsion of heavy metals, because most insects are exposed to heavy metal stress through their diet. The main components of insect digestion and absorption occurs in the midgut, where heavy metals primarily accumulate, and have the greatest impact on the insect midgut [13,14]. The uptake and accumulation of heavy metals in the insect midgut are primarily mediated by midgut epithelial cells, which contain many metal particles capable of adsorbing surplus heavy metal ions. Heavy metals deposited by these cells are subsequently excreted as intestinal metabolites during molting [15]. Simultaneously, midgut epithelial cells can secret a lamellar tubular membrane structure, the peritrophic membrane (PM), to resist the harm of pesticides, heavy metals, and pathogens [16]. The primary component of PM in the midgut is chitin, which makes the biosynthesis, assembly, and degradation of PM intimately associated with chitin synthesis and metabolism [17]. In insects, chitin is synthesized using trehalose as the initial substrate and converted through a sequential enzymatic pathway involving trehalase (TRE), hexokinase (HK), glucose-6-phosphate isomerase (G6PI), fructose-6-phosphate aminotransferase (GFAT), glucosamine-6-phosphate N-acetyltransferase (GNPNA), phosphoacetylglucosamine mutase (PAGM), UDP-N-acetylglucosamine pyrophosphorylase (UAP), and chitin synthase (CHS) [18]. CHS is divided into two types, CHS-A and CHS-B [19]. *Chitin synthase-2* (*CHS-2*), which encodes CHS-B, is highly expressed only in insect midgut epithelial cells and is responsible for synthesizing chitin in the peritrophic membrane [20]. Previous studies demonstrated the indispensability of *CHS-2* in forming PM within the midgut of *Ae. albopictus*. When *CHS-2* is silenced, the fourth-instar of *Ae. albopictus* cannot generate a complete PM structure [21].

Furthermore, metallothionein (MT) and heat shock proteins (HSPs) are crucial mechanisms that enable insects to alleviate heavy metal stress. MT, a class of binding proteins, can form chelates with metal ions, thereby reducing or eliminating the toxic effects of heavy metals. As molecular chaperones, HSPs can restore the functional structure of damaged proteins, thereby exerting cytoprotective effects [22]. Recently, a growing body of research has explored the potential of MT and HSPs as biomarkers of heavy metal pollution [23,24]. Heavy metals can generate a multitude of reactive oxygen species (ROS) after entering the insect body, triggering redox reactions and subsequent activation of a cascade of antioxidant defense enzymes, including superoxide dismutase (SOD), peroxidase (POD), catalase (CAT) and glutathione peroxidase (GSH-PX), which are very sensitive to environmental pollutants. These enzymes have been used as biomarkers in ecotoxicological studies [25].

*Ae. albopictus*, belongs to the family Diptera and is a vital viral vector capable of transmitting various pathogens, including dengue and yellow fevers. The eggs, larvae, and pupae of *Ae. albopictus* grow and develop in aquatic environments, whereas adult mosquitoes reside on land. The larvae of *Ae. albopictus*, which reside in water and subsist on algae, protozoa, and yeast, exhibit a certain capacity to accumulate heavy metals. Excessive heavy metal infiltration may have detrimental effects on the growth, development, and reproduction of *Ae. albopictus* [26]. However, the mechanisms underlying the response to *Ae. albopictus* to heavy metal stress, particularly the response of *CHS-2* to heavy metal stress and its potential functions and regulatory pathways, remains elusive. The lethal concentration 50% (LC50) of cadmium for *Ae. albopictus*, as determined in laboratory conditions, was 263 mg/L. In the present study, the insect vector *Ae. albopictus* was first exposed to various concentrations of Cd to investigate the effects of different Cd stress levels on the chitin synthesis and metabolism of *Ae. albopictus*. Subsequently, the *CHS-2* was silenced via RNA interference (RNAi) and combined with three Cd stress concentrations to explore the response mechanism and potential adaptive functions of *CHS-2* under Cd pollution stress. This clarifies the resistance adaptation and internal expression mechanisms of *Ae. albopictus* under Cd stress. This study supports the morphological changes in the midgut PM of *Ae. albopictus* larvae as potential biomarkers of environmental Cd pollution. Simultaneously, the *CHS-2* gene is considered to be very important for the development of resistance to *Ae. albopictus.*

## 2. Materials and Methods

### 2.1. Mosquitoes

*Ae. albopictus* mosquitoes, sourced from Sun Yat-sen University (Guangdong Province, China), were maintained and bred through more than three generations under controlled conditions. These conditions included a temperature of 27 ± 1 °C, 70% relative humidity, and a light/dark cycle of 14:10 h, within an artificial climate chamber. The cat food was ground into powder and mixed with yeast powder at a ratio of 2:1, then fed to the first- and second-instar larvae. For the third- and fourth-instar larvae, cat chow powder alone was provided [27]. Adult mosquitoes, after eclosion, were sustained with a solution of 10% sugar water. Mice were utilized for mosquito blood-feeding to facilitate oviposition.

### 2.2. Cd Stress on Ae. albopictus

According to the national standard GB/T 602-2002 [28], a Cd solution (1 g/L) was prepared using CdCl_2_·$\frac{5}{2}$H_2_O, and successively diluted to concentrations of 0, 6.25, 12.5, 25, 50, 100, 200, 300, and 400 mg/L. Fifty fourth-instar larvae of approximately the same size were selected and placed in each cup for exposure, and the 12 h mortality rate was recorded. The concentration of Cd solution corresponding to 50% mortality was determined to be 263 mg/L using probit regression analysis. Based on the semi-lethal concentration and the results of previous laboratory experiments, Cd stress concentrations (0, 25, 50, and 100 mg/L) were selected, and larvae treated with 0 mg/L were used as negative controls. The fourth-instar larvae were exposed to the above concentrations of Cd solution, and samples were taken at 12, 24 and 36 h after exposure.

### 2.3. Microinjection

Using the successfully sequenced *CHS-2* intermediate fragment as a template, T7 promoter sequences were added to the 5′ end of primers (Table 1) [21]. Double-stranded RNA (dsRNA) was synthesized using a T7 RiboMAXTM Express RNAi System kit (LABOOT, Hangzhou, China) and purified, and the specific experimental steps were in accordance with the instructions. The same method was used to synthesize dsRNA using the *green fluorescent protein* (*GFP*) as a negative control. Seven hundred and fifty well-developed fourth-instar larvae of the same size were randomly selected for microinjection. The ds*CHS-2* (200 ng) was dissolved in 100 nL of water and injected into the soft spot between the penultimate and third-last body segments of the larvae using a microinjection device. The same method was used to inject ds*GFP* into another group of 750 fourth-instar larvae. The expression levels of the *CHS-2* gene in *Ae. albopictus* larvae were reduced by 50% at both 12 h and 24 h post-injection of ds*CHS-2* [21].

### 2.4. Gene Expression Analysis

Ten fourth-instar larvae of *Ae. albopictus* were collected each treatment concentration after 12 h of Cd treatment. Total RNA was extracted using the TRIzol method, and the quality and concentration were detected using agarose gel electrophoresis and microspectrophotometry (Thermo, Waltham, MA, USA). cDNA was synthesized using the Prime ScriptTM RT Reagent Kit (Haofeng, Hangzhou, China) according to the manufacturer’s instructions. Through the National Center for Biotechnology Information and relevant literature, chitin synthesis genes *HK*, *G6PI*, *GFAT*, *GNPNA*, *PAGM*, *UAP*, and *CHS-2*, as well as chitin metabolism enzyme genes *chitinase2* (*Cht2*) and *chitinase10* (*Cht10*), were selected. Primer premier 5.0 software was used to design qPCR primers (Table 2), and the expression levels were measured using qPCR. The qPCR reaction mixture (10 µL) contained 5 µL of TB Green ^®^ Premix Ex Taq ™, 3.2 µL of ddH_2_O, 0.4 µL each of upstream and downstream primers and 1 µL cDNA. The qPCR procedure was as follows: 10 min at 94 °C; 30 s at 94 °C, 30 s at 59 °C, 45 s at 72 °C for a total of 30 cycles, and then 10 min at 72 °C. A default dissolution curve analysis was performed. The *actin* gene was used as an internal control, and the 2^−ΔΔCT^ method was used to calculate the relative expression of genes related to chitin synthesis and metabolism after Cd treatment for 12 h.

### 2.5. Determination of Chitin Content in the Midgut

The midguts from 45 *Ae. albopictus* mosquitoes per group, exposed to different concentrations of cadmium for 12 h, were dissected and collected. The content of chitin in the midgut of larvae was determined with reference to the method of Liu et al. [29], and the principle is that chitinase hydrolyzes chitin to produce N-acetylglucosamine, and the intermediate compound produced by the cross-reaction of N-acetylglucosamine and alkali can further react with p-dimethylaminobenzaldehyde to produce chromogenic substances.

### 2.6. Antioxidant Enzyme Activity Assay

At 12, 24, and 36 h after dsRNA injection, 45 fourth-instar larvae exposed to four concentrations of Cd were collected, respectively. Then 0.9% normal saline was added according to the ratio of weight (g):volume (mL) = 1:9, and the supernatant was taken after ice bath breaking. SOD (Cat. No. A001-1-2) activity was measured by xanthine and xanthine oxidase reaction system using Kit (Jiancheng, Nanjing, China). POD (Cat. No. A084-3-1), CAT (Cat. No. A007-2-1) and GSH-PX (Cat. No. A005-1-2) activities were measured according to the principle of catalyzing H_2_O_2_ using Kit (Jiancheng, Nanjing, China), and the specific experimental steps were performed in accordance with the manufacturer’s instructions.

### 2.7. Observation of the PM of the Midgut

The fourth-instar larvae exposed to four concentrations of Cd for 12 h and the fourth-instar larvae injected with dsRNA and exposed to 50 mg/L Cd for 12, 24 and 36 h were used as materials, and the head and tail were removed according to Gregor et al. production, sectioning and staining methods [30]. After fixation, the larvae were embedded in paraffin using a paraffin embedding machine (MICROM, Walldorf, Germany) and cut into 5-µm-thick sections. After dewaxing and cleaning, the samples were stained with hematoxylin-eosin staining (HE staining). A microscope camera system (Axio Observer A1+Stemi2000, ZEISS, Oberkochen, Germany) was used for photograph and observation.

### 2.8. Data Analysis

IBM SPSS Statistics 23.0 was used for the statistical analysis. Data on gene expression, enzyme activity, and chitin content were analyzed using one-way ANOVA and Duncan’s test. Mortality data were analyzed using the chi-square test (*p* < 0.05 indicated a significant difference, annotated with “*”; *p* < 0.01 indicated a significant difference, annotated with “**”). Graphs were created using SigmaPlot 10.0. The normality and homogeneity of the data were assessed using three replicates for each treatment concentration and tissue sample quantity.

## 3. Results

### 3.1. Expression Changes in Genes Related to Chitin Metabolism Pathway in Ae. albopictus After Cd Stress

**Figure 1:**
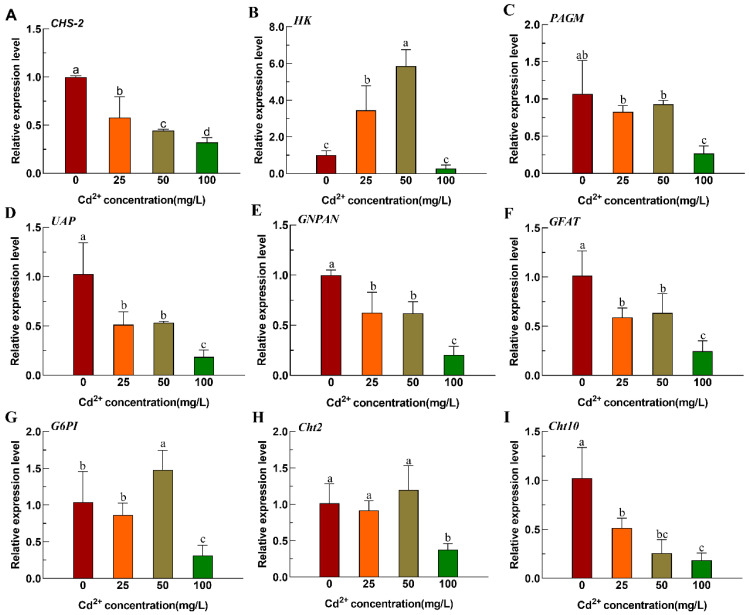
Relative expression of chitin metabolic pathway-related genes of *Ae. albopictus* after Cd stress. (**A**) *CHS-2*. (**B**) *HK*. (**C**) *PAGM*. (**D**) *UAP*. (**E**) *GNPAN*. (**F**) *GFAT*. (**G**) *G6PI*. (**H**) *Cht2*. (**I**) *Cht10*. Values represent mean ± SE. Different lowercase letters above the bar indicate that the difference is statistically significant. Relative expression levels were calculated in comparison with that of the fourth-instar larvae in 0 mg/L Cd solution, which was ascribed an arbitrary value of 1.

Acute Cd stress affects the expression of genes related to chitin synthesis and metabolism in the fourth-instar larvae of *Ae. albopictus*. The expression of *CHS-2* gradually decreased with increasing Cd concentration. Significant inhibition of *CHS-2* expression was observed at a Cd concentration of 100 mg/L (Figure 1A). Similarly, the expression of genes associated with chitin synthesis, including *PAGM*, *UAP*, *GNPNA*, and *GFAT*, was inhibited by a pronounced decrease in their expression levels at a Cd concentration of 100 mg/L (Figure 1C–F). Under low Cd stress (50 mg/L), the expression of *HK* and *G6PI* increases, whereas it significantly decreased under 100 mg/L Cd stress (Figure 1B,G). The expression of the chitin metabolism enzyme *Cht2* remained unchanged at low-Cd concentrations and significantly decreased at 100 mg/L (Figure 1H). The relative expression of *Cht10* also decreased with increasing Cd concentrations (Figure 1I).

### 3.2. Determination of Chitin Content and Observation of Midgut Structure of Aedes albopictus Larvae Under Cadmium Stress

**Figure 2:**
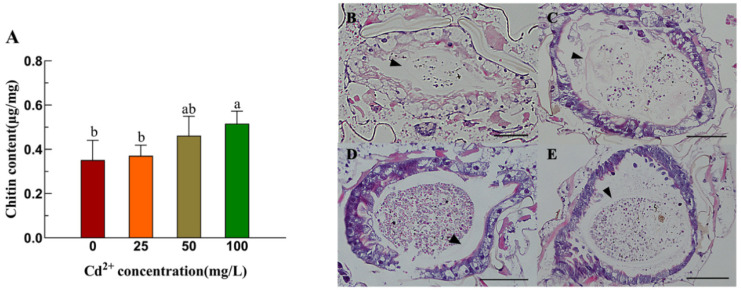
After different concentrations cadmium stresses of *Ae. albopictus* larva of chitin content change and intestine structure observation. (**A**) Chitin content in the midgut of the fourth-instar larvae of *Ae. albopictus* after 12 h of Cd stress. Values represent mean ± SE. Different lowercase letters above the bar indicate that the difference is statistically significant. Fourth-instar larvae 12 h exposed to Cd solutions of different concentrations were used as materials, and the head and tail were removed, embedded in paraffin, sectioned, stained with HE, and observed under a microscope. (**B**) 0 mg/L, (**C**) 25 mg/L (**D**) 50 mg/L (**E**) 100 mg/L. The scale indicates 100 μm. The triangle indicates the position of the PM.

After Cd stress, the chitin content in the midgut of fourth-instar larvae of *Ae. albopictus* demonstrated a significant positive correlation with Cd concentration. Under 100 mg/L Cd stress, the chitin content notably increased (Figure 2A). Compared with the control group (Figure 2B), the midgut PM of the fourth-instar *Ae. albopictus* larvae were clearer and continuous after Cd stress, and the higher the concentration of Cd, the more obvious the PM thickening (Figure 2C–E).

### 3.3. Morphological Changes in the Midgut of Ae. albopictus Under Cd Stress After CHS-2 Silencing

**Figure 3:**
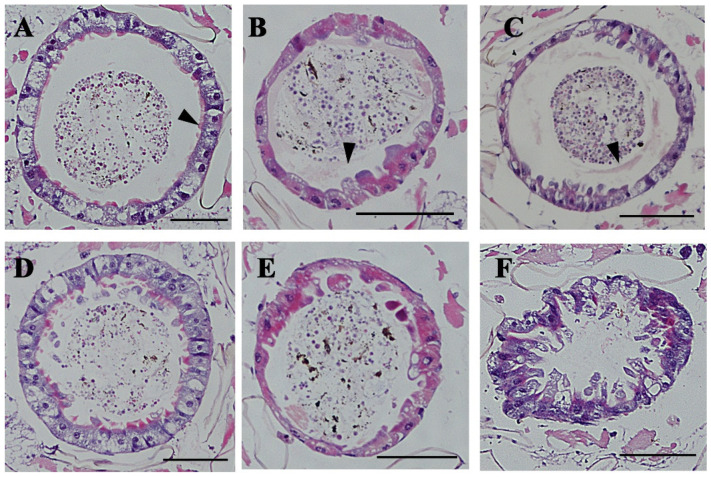
Slices of the fourth-instar larvae of *Ae. albopictus* after *CHS-2* silencing combined with 50 mg/L Cd stress. After injection of dsRNA, fourth-instar larvae were exposed to 50 mg/L Cd solution for 12, 24 and 36 h, were used as materials, and the head and tail were removed, embedded in paraffin, sectioned, stained with HE, and observed under a microscope. (**A**) 12 h after ds*GFP* injection. (**B**) 24 h after ds*GFP* injection. (**C**) 36 h after injection of ds*GFP*. (**D**) 12 h after injection of ds*CHS-2*. (**E**) 24 h after injection of ds*CHS-2*. (**F**) 36 h after injection of ds*CHS-2*. The scale is 100 μm, and the black arrow in the figure is the normal PM.

Under 50 mg/mL cadmium stress, the PM of the larvae in the ds*GFP* group was visible (Figure 3A,B). With increased exposure time to cadmium, the epithelial cells in the larvae midgut exhibited a slight deformity (Figure 3C). In contrast, the larval PM in the ds*CHS-2* group was destroyed, and the epithelial cells were severely deformed after exposure to the Cd solution for only 12 h (Figure 3D). After 24 h, a large number of epithelial cells were convex, and some epithelial cells were vacuolized (Figure 3E). However, after exposure to a cadmium solution for 36 h, the whole midgut was severely deformed and could not maintain its normal morphology (Figure 3F).

### 3.4. Mortality of Ae. albopictus After CHS-2 Silencing Combined with Cd Stress

**Figure 4:**
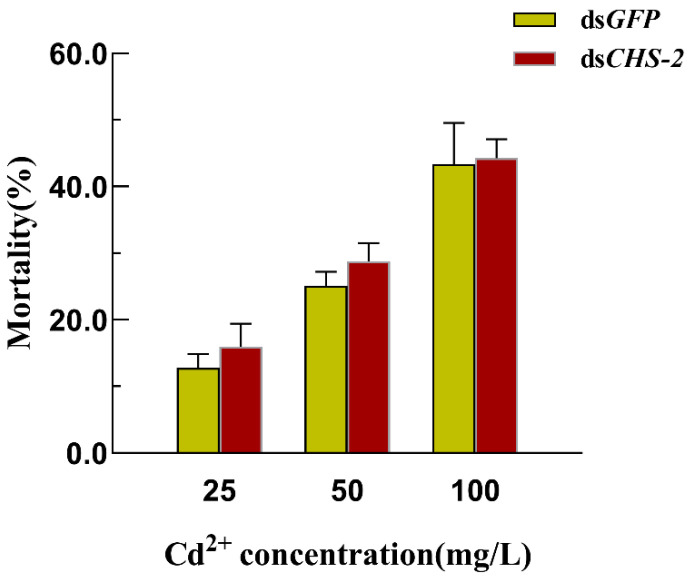
Mortality of *Ae. albopictus* after *CHS-2* silencing combined with acute Cd stress. Values represent mean ± SE.

The findings of previous studies indicate that silencing the *CHS-2* alone for 24 h has no discernible impact on the larval survival of *Ae. albopictus* [21]. However, the application of Cd for 12 h after RNA interference caused a significant increase in larval mortality, with higher Cd concentrations correlated with increased mortality. The mortality rate in the ds*CHS-2* injected group following Cd stress was virtually indistinguishable from that of the control group, indicating no significant disparity (Figure 4).

### 3.5. Changes in HSP70 and MT Expression in Ae. albopictus After CHS-2 Silencing Combined with Acute Cd Stress

**Figure 5:**
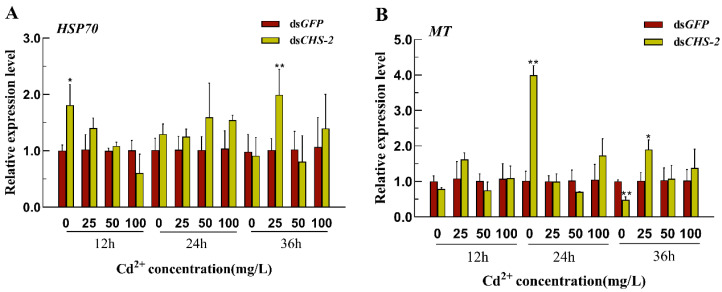
The relative expression of *HSP70* and *MT* in *Ae. albopictus* after *CHS-2* silencing combined with Cd acute stress. (**A**) *HSP70*. (**B**) *MT*. Relative expression levels were calculated in comparison with ds*GFP* exposed to the same concentration of Cd solution for the same time, which was ascribed an arbitrary value of 1. Values represent mean ± SE. (One-way ANOVA, Duncan test, * *p* < 0.05, ** *p* < 0.01).

The expression of *HSP70* was significantly upregulated within 12 h following silencing of *CHS-2*, returning to basal levels at 24 and 36 h (Figure 5A). The expression of *MT* initially increased and then declined after *CHS-2* silencing (Figure 5B). Under low-Cd stress (25 mg/L), the expression levels of *HSP70* and *MT* were significantly upregulated after 36 h.

### 3.6. Changes in the Activities of Antioxidant Enzymes in Ae. albopictus After CHS-2 Silencing Combined with Cd Stress

**Figure 6:**
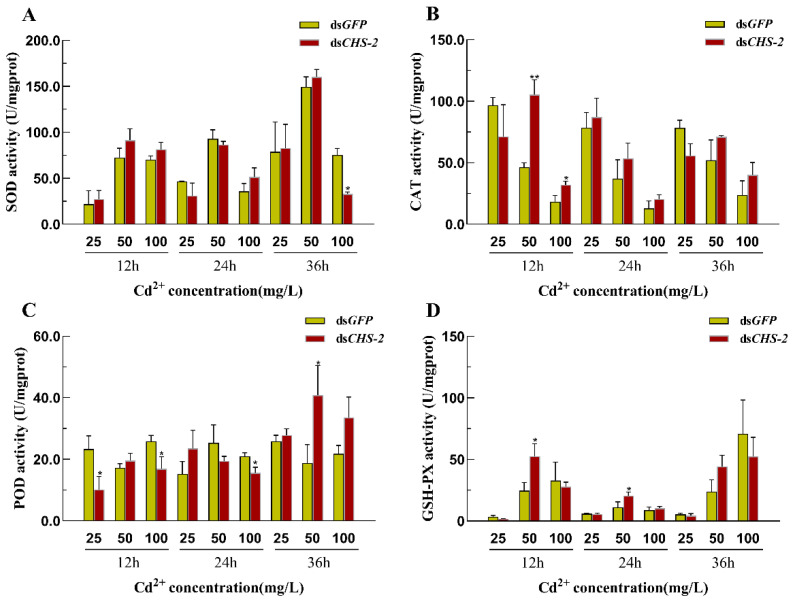
Activity of antioxidant enzymes in *Ae. albopictus* after *CHS-2* silencing combined with Cd stress. (**A**) SOD. (**B**) CAT. (**C**) POD. (**D**) CSH-PX. Values represent mean ± SE. (One-way ANOVA, Duncan test, * *p* < 0.05, ** *p* < 0.01).

The combination of *CHS-2* silencing and Cd stress partially influenced the activity of antioxidant enzymes in *Ae. albopictus*. Compared with the ds*GFP* group, significant down-regulation of SOD was observed only 36 h after 100 mg/L Cd stress (Figure 6A). CAT activity significantly increased within a brief period and subsequently returned to that of the control group (Figure 6B). POD activity was prominently down-regulated at 12 h post-25 mg/L Cd stress and 12 and 24 h after 100 mg/L Cd stress; however, it was significantly up-regulated at 36 h following 50 mg/L Cd stress (Figure 6C). GSH-PX activity was significantly up-regulated at 12 and 24 h post-50 mg/L Cd stress (Figure 6D).

## 4. Discussion

Cd exposure has numerous detrimental effects. For example, Cd stress has been observed to decrease the survival and reproductive rates of *Anopheles gambiae* [31] and impede the cell proliferation of *Ae. albopictus* [32], and more. Previous studies have revealed that prolonged Cd stress can result in smaller larvae and pupae, suppress pupation and emergence rates, extend the developmental cycle of *Ae. albopictus*, and negatively affect the reproductive success of adult *Ae. albopictus* [26]. The insect midgut is the main target organ for heavy metal stress, and midgut epithelial cells secrete a reticular structure, the PM, to resist heavy metal damage. Proteins, mucopolysaccharides and chitin are the main components of the PM. Therefore, *CHS-2* and its PM chitin may play important roles in the response of aquatic insects to heavy metal stress, and the synthesis and metabolism of trehalose and chitin mediated by *CHS-2* may play an important role in the adaptation of aquatic insects to heavy metal pollution. *CHS-2* has previously been identified as predominantly expressed in the midgut of *Ae. albopictus* larvae and plays a pivotal role in PM formation. Upon silencing *CHS-2*, a significant reduction in chitin content was observed in the midgut of fourth-instar larvae of *Ae. albopictus*, leading to the partial or complete destruction of the PM [21]. Abedi and Brown found large amounts of dichlorodiphenyltrichloroethane (DDT) in the larval PM of *Aedes aegypti* mosquitoes, which are resistant to the insecticide DDT [33]. In the present study, except for *HK* and its downstream *G6PI*, the expression of genes associated with chitin synthesis and metabolic pathways in *Ae. albopictus* were suppressed under Cd stress (Figure 1). Lin et al. demonstrated that *HK* is a crucial gene regulating ROS in insects and that reducing *HK* expression increases ROS activity [34]. Heavy metals can generate substantial amounts of ROS in insects; thus, high HK expression at low concentrations might mitigate ROS activity (Figure 1B). As the expression of *HK* increased, its downstream gene *G6PI* also increased (Figure 1G). In contrast to the low expression of chitin synthesis genes, chitin content in the larval midgut was significantly increased (Figure 2A). This discrepancy might be attributed to the post-transcriptional regulation of *CHS-2* [35]. The midgut of the fourth-instar larvae was examined for varying concentrations Cd-induced stress using HE staining and paraffin sections. These findings indicate that the midgut epithelial cells of *Ae. albopictus* fourth-instar larvae exhibited vacuolization under Cd stress accompanied by significant PM thickening (Figure 2C–E). This thickening was more pronounced at higher Cd concentrations, which concurred with the increased chitin content in the midgut. Wu et al. observed a similar phenomenon in the midgut of *Boettcherisca peregrina* larvae following Cd stress [36]. Based on the aforementioned results, we postulate that Cd stress could prompt the midgut to secrete a substantial amount of PM to facilitate the metabolism of excess Cd, subsequently leading to an increase in chitin content.

To cope with the stress caused by heavy metals, insects have evolved various adaptive mechanisms, including active heavy metal deposition and excretion, initiation of oxidative stress responses, and regulation of *MT* and *HSP* expression. First, the midgut of *Ae. albopictus* fourth-instar larvae exposed to 50 mg/L of Cd after silencing *CHS-2* was observed. When *CHS-2* were silenced, the midgut of *Ae. albopictus* larvae failed to develop a complete PM, thereby losing its protective effects. In comparison to simple Cd stress (Figure 2D), the vacuolization of midgut epithelial cells was exacerbated and progressively deformed as the duration of Cd stress increased (Figure 3F), which was consistent with the results of Dabor et al.’s study on the alterations in bee midgut cells induced by CdO and PbO [37]. Subsequent analysis of larval mortality revealed a positive correlation between the mortality of *Ae. albopictus* larvae and Cd concentrations, whereas the silencing *CHS-2* did not significantly influence larval mortality under Cd stress (Figure 4). In previous experiments, silencing the *CHS-2* gene alone did not affect the survival of *Ae. albopictus* larvae [21]. This suggests *Ae. albopictus* larvae cope with excessive Cd stress via alternative mechanisms following the loss of PM deposition metabolic functions. MT has been identified as the primary ligand of Cd in numerous organisms and is associated with biological tolerance to Cd [38,39]. MT is mainly distributed in the digestive tract of insects, and the Cd ingested by fleshfly larvae mostly combines with proteins to form MT in the digestive tract [40]. The induction of HSPs is a crucial mechanism insects use to counteract heavy metal stress. The expression of HSPs serves as a biological indicator of oxidative stress induced by heavy metals. The HSP70 family is particularly sensitive to Cd stress, and the expression of *HSP70* in insects is significantly upregulated following Cd exposure [41,42]. In this study, the combination of *CHS-2* silencing and Cd stress occasionally altered the expression of *MT* and *HSP70*. These changes appeared irregular. Given that some members of the HSP family are constitutively expressed, we speculate that the selected genes may not be directly related to cadmium stress. The precise underlying mechanisms warrant further investigation.

The ingestion of heavy metals by insects generates a multitude of reactive oxygen free radicals, which in turn stimulate the activation of a panel of antioxidant defense enzymes, including SOD, POD, CAT, and GSH-PX. SOD rapidly disintegrates ROS into H_2_O_2_ and concurrently activates CAT and POD to further decompose H_2_O_2_ into H_2_O and CO_2_ [43]. GSH-PX converts oxidized glutathione to its reduced form and aids in decomposing a portion of H_2_O_2_ [44]. It has been found that insect PM has a stronger oxidative stress response than other tissues and can secrete antioxidant enzymes, such as SOD, to counteract free radical damage [45,46]. Uncharged chitin and positively charged proteoglycans in insect PM can chelate heavy metal ions, thereby partially mitigating the damage induced by oxidative stress. Under low Cd stress, antioxidant enzyme activity is f activated in *Oxya chinensis*, whereas it is inhibited under high-Cd stress [47]. In the present study, the antioxidant enzymes activity in *Ae. albopictus* under Cd stress following *CHS-2* silencing was investigated. Post-silencing of *CHS-2*, SOD activity remained unaltered under Cd stress, but exhibited a significant down-regulation only 36 h after 100 mg/L Cd stress (Figure 6A). The overproduction of hydrogen peroxide free radicals, triggered by strong oxidative stress reactions following the inhibition of *CHS-2*, was speculated to oxidize cysteine residues in SOD, leading to unchanged or even decreased SOD activity [48]. Significant increases in the activities of CAT and POD were observed under both short-term (12 h) and long-term (36 h) Cd stress conditions following the silencing of *CHS-2* (Figure 6B,C), suggesting that these enzymes substituted for PM in response to oxidative stress induced by Cd at different timepoints. POD activity was significantly reduced within 24 h of *CHS-2* silencing. Ma et al. found that POD affects cuticle formation in *Cyprinus carpio* [49], and PM is composed of cuticle proteins in addition to chitin [50]. Thus, we postulate that silencing *CHS-2* leads to a failure in midgut PM formation, diminishing the requirement for cuticular proteins and, consequently, POD activity.

In conclusion, Cd stress can influence chitin synthesis and metabolism in fourth-instar larvae of *Ae. albopictus*, simultaneously promoting the accumulation of excess Cd in larval midgut PM. Upon silencing *CHS-2*, a key gene for chitin synthesis, fourth-instar larvae of *Ae. albopictus* failed to develop a complete PM, affecting Cd deposition and metabolism in the midgut and triggering a robust oxidative stress response. Thus, *CHS-2* modulates the formation of *Ae. albopictus* intestinal PM, which is involved in Cd-induced oxidative stress. The PM is a semi-permeable tissue composed of chitin and proteins, offering protection against pathogens and macromolecular compounds, thus the anti-stress ability of *Ae. albopictus* was enhanced, who live long time in the Cd-polluted water environment, and then leading to worries of their population expansion. In the future, our research will focus on the following three points: First, we will observe the morphological changes in the PM in *Ae. albopictus* larvae under Cd stress in more detail, and try to use it as a potential biological indicator for environmental monitoring; Second, the stress resistance of *Ae. albopictus* was tested through long-term or multiple generations of Cd stress, revealing that environmental pollution may imperceptibly improve the resistance of pests, thus increasing the difficulty and cost of prevention and control, and increasing the public’s attention to environmental pollution. Third, the function and adaptation mechanisms of *CHS-2* in *Ae. albopictus*’ response to long-term Cd stress, including its regulatory relationship with related antioxidant enzyme genes, was explored to clarify the resistance mechanism of *Ae. albopictus* and attempt to control *Ae. albopictus* by using resistance or other control methods.

## Figures and Tables

**Table 1:** Primers for synthesis of dsRNA.

Primer Name	Forward Primer (5′-3′)	Reverse Primer (5′-3′)	NCBI
ds*CHS-2*	GGATCCTAATACGACTCACTATAGGAGTCAACGCACCGTACAGGA	GGATCCTAATACGACTCACTATAGGGAAGAGCAACACCAAACCGA	LOC109405983
ds*GFP*	GGATCCTAATACGACTCACTATAGGAAGGGCGAGGAGCTGTTCACCG	GGATCCTAATACGACTCACTATAGGCAGCAGGACCATGTGATCGCGC	XM_013480425.1

The underlined is the T7 promoter.

**Table 2:** Primers for qRT-PCR.

Primer Name	Forward Primer (5′-3′)	Reverse Primer (5′-3′)	NCBI
*actin*	GCTACGTCGCCCTGCACTT	AGGAACGACGGCTGGAAGA	DQ657949.1
*CHS-2*	GGAGACCAAAGGATGGGACG	CCTGTAAGGACGATGACGAATGT	XM_019679038.3
*Cht2*	GACTCGGATGACAAGGGTTT	CAGGGCTTCACATACTTCGTT	XM_029879282.1
*Cht10*	GCCACGGATACTTAGAATAGCG	CTGTTTGACGGTCGTTGATTT	XM_029869372.1
*HK*	GGGAGAAGCCAGCGAAGATA	GATGTCTGCTTCGGGATGTG	AY705876.1
*PAGM*	GCCATTTCTGACATGCTACT	CGTTTCGGTCTTCTACTTTGA	XM_019671844.2
*UAP*	AGTGCTCTATTTACACGCTCAT	ACTCCGACCGCTTCATTT	GAPW01001510.1
*GNPNA*	CAGTCCTCCCATTTCAGCC	AACGAAACATCGCCCACC	XM_019707567.2
*GFAT*	TCAACGGGCAACATCCAG	AAGCGTCCGATGCAAAGA	XM_019671518.2
*G6PI*	GGAGGATGACATTCGCTTCG	CGGTGATACGGTTCTTGGAG	XM_019673634.3
*HSP70*	GATGTGTCCCTTTTGACCATT	CGACTTCACGACGCAGTTTCT	JN132154.1
*MT*	ATGCCTTGCCCATGCGA	CAGTCAGATCCGCAGTTGC	

## Data Availability

The original contributions presented in this study are included in the article. Further inquiries can be directed to the corresponding authors.
